# Evolving epidemiology, clinical features, and genotyping of dengue outbreaks in Bangladesh, 2000–2024: a systematic review

**DOI:** 10.3389/fmicb.2024.1481418

**Published:** 2024-10-30

**Authors:** Nadim Sharif, Rubayet Rayhan Opu, Tama Saha, Abdullah Ibna Masud, Jannatin Naim, Khalaf F. Alsharif, Khalid J. Alzahrani, Eduardo Silva Alvarado, Irene Delgado Noya, Isabel De la Torre Díez, Shuvra Kanti Dey

**Affiliations:** ^1^Department of Microbiology, Jahangirnagar University, Dhaka, Bangladesh; ^2^Department of Clinical Laboratories Sciences, College of Applied Medical Sciences, Taif University, Taif, Saudi Arabia; ^3^Universidad Europea del Atlántico, Santander, Spain; ^4^Universidad Internacional Iberoamericana, Campeche, Mexico; ^5^Universidad Internacional Iberoamericana, Arecibo, PR, United States; ^6^Universidad de La Romana, La Romana, Dominican Republic; ^7^Universidade Internacional do Cuanza, Cuito, Bié, Angola; ^8^Fundación Universitaria Internacional de Colombia, Bogotá, Colombia; ^9^University of Valladolid, Valladolid, Spain

**Keywords:** dengue, outbreak, epidemiology, seasonality, Bangladesh

## Abstract

**Background:**

The 2023 dengue outbreak has proven that dengue is not only an endemic disease but also an emerging health threat in Bangladesh. Integrated studies on the epidemiology, clinical characteristics, seasonality, and genotype of dengue are limited. This study was conducted to determine recent trends in the molecular epidemiology, clinical features, and seasonality of dengue outbreaks.

**Methods:**

We analyzed data from 41 original studies, extracting epidemiological information from all 41 articles, clinical symptoms from 30 articles, and genotypic diversity from 11 articles. The study adhered to the standards of the Preferred Reporting Items for Systematic Review and Meta-Analysis (PRISMA) Statement and Cochrane Collaboration guidelines.

**Results:**

A total of 565,438 dengue cases and 2,587 fatalities were documented from January 2000 to March 2024. Notably, 60% of cases during the 2019 and 2023 outbreaks were reported in regions previously considered non-endemic. Fatalities were more frequent among women (70%). The majority of the studies (95–100%) used the NS1Ag test, followed by IgG or IgM and RT-PCR tests. New hotspots of dengue transmission were identified in the southern (Khulna, 10.8% and Barishal, 11.8%) and southeastern (Chattogram, 13.8%) regions of Bangladesh. Serotyping was conducted on 92.4% (1,456 of 1,575) of isolates between 2012 and 2023. Of the four serotypes, DENV3 was the most prevalent (57%), followed by DENV2 (30%), DENV1 (11%), and DENV4 (<1%). Genotype DENV3-I (43 of 59 isolates) was the most prevalent, followed by DENV3-II (8 of 59). The highest frequency of dengue cases was observed in August (26.3%), followed by September (22.5%), October (20.2%), and November (13.08%). Fever (90.51, 95% CI 85–100%) was the most prevalent symptom, followed by headache (57.98, 95% CI 12–100%), vomiting (51.16, 95% CI 23–91%), abdominal pain (34.12, 95% CI 12–85%), and myalgia (25.53, 95% CI 13–85%), respectively.

**Conclusion:**

This study provides integrated insights into the molecular epidemiology, clinical features, seasonality, and transmission of dengue in Bangladesh and highlights research gaps for future studies.

## Introduction

Dengue is an acute febrile disease caused by the dengue virus, which can now spread to more than 125 countries worldwide (WHO Dengue fact sheet, 2024). According to the World Health Organization (WHO), confirmed cases of dengue have increased from 505,430 in 2000 to 5. 2 million in 2019 (WHO Dengue fact sheet, 2024; Hadinegoro, 2012; Health bulletin on current Dengue situation published by DGHS, 2024; WHO, 2024; Deen et al., 2006; Prattay et al., 2022). It is estimated that 300 million cases of dengue occur, with 100 million cases reported clinically every year worldwide. Furthermore, 4 billion people are at risk of contracting dengue (WHO Dengue fact sheet, 2024; Health bulletin on current Dengue situation published by DGHS, 2024; Guo et al., 2017; Prattay et al., 2022). The perception that dengue is confined to tropical and subtropical areas is changing, as recent global expansion of cases has been documented (Health bulletin on current Dengue situation published by DGHS, 2024; WHO, 2024; Deen et al., 2006; Prattay et al., 2022; Muraduzzaman et al., 2018).

The dengue virus is transmitted from human to human by *Aedes* spp. mosquitoes. However, in the last decade, these vectors have rapidly spread to distant regions beyond the traditional endemic areas, becoming established in regions previously unexposed to dengue (WHO, 2024; Islam et al., 2022b; Ahmed et al., 2016). Rapid transportation, increased travel, and the mosquitoes’ adaptation to new environments have significantly contributed to this widespread transmission.

Bangladesh is classified as an endemic region for dengue fever (Prattay et al., 2022; Muraduzzaman et al., 2018; Islam et al., 2022b; Ahmed et al., 2016; Islam et al., 2019; Islam et al., 2022a; Khan et al., 2021; Hasan et al., 2021a; Yang et al., 2023; Rafi et al., 2020). The first case of dengue was reported in early 2000, and outbreaks have been reported regularly in Bangladesh since 2000 (Health bulletin on current Dengue situation published by DGHS, 2024; WHO, 2024; Deen et al., 2006; Prattay et al., 2022; Islam et al., 2022a; Khan et al., 2021; Hasan et al., 2021a; Yang et al., 2023; Rafi et al., 2020; Shultana et al., 2019). However, the lack of strong and effective surveillance has likely led to an underestimation of the true number of cases and fatalities. Recent larger outbreaks, including 101,500 cases in 2019 and 321,073 cases in 2023, have spread across the majority of the non-endemic regions in Bangladesh, posing a major health threat (Hossain et al., 2023; Health bulletin on current Dengue situation published by DGHS, 2024; Rafi et al., 2020; Shultana et al., 2019; Mahmood et al., 2021; Yesmin et al., 2023b; Rahim et al., 2023). After COVID-19, dengue has emerged as a major public health issue, with 1,705 people dying from the outbreak in 2023 (Health bulletin on current Dengue situation published by DGHS, 2024; WHO, 2024). Many non-endemic regions have now become hotspots of dengue transmission. Without effective treatment and prevention measures, the ongoing spread of dengue has become an alarming global health concern (Ahmed et al., 2016; Hasan et al., 2021a).

The dengue virus is a single-stranded, positive-sense RNA virus with a genome of approximately 11,000 bases (Prattay et al., 2022; Muraduzzaman et al., 2018; Islam et al., 2022b). The genome encodes three structural proteins—capsid protein (C), membrane protein (M), and envelope protein (E)—as well as seven non-structural proteins: NS1, NS2a, NS2b, NS3, NS4a, NS4b, and NS5. Dengue virus has been classified into four serotypes: DENV-1, DENV-2, DENV-3, and DENV-4. Based on the genetic and phylogenetic analysis, each serotype is further divided into different genotypes (WHO Dengue fact sheet, 2024; WHO, 2024; Islam et al., 2006; Aziz et al., 2002; Sultana et al., 2020; Hossain et al., 2003). However, genotypic surveillance data for the dengue virus is significantly lacking in Bangladesh (Health bulletin on current Dengue situation published by DGHS, 2024; Prattay et al., 2022; Amin et al., 2022; Kabir et al., 2020).

According to the WHO dengue case classification (WHO, 2009), cases of dengue can be classified as dengue without warning signs, dengue with warning signs, and severe dengue (Hadinegoro, 2012). Symptomatic infection can manifest as undifferentiated fever (viral syndrome), dengue fever syndrome (DFS), or dengue hemorrhagic fever (DHF). In many cases of DFS, unusual hemorrhaging may be present, while some DFS cases occur without hemorrhaging. DHF can be further classified into cases with or without shock, with dengue shock syndrome (DSS) representing the more severe form (Hadinegoro, 2012; Deen et al., 2006).

The majority of the cases are reported to be mild, with asymptomatic or mildly symptomatic infections. Common symptoms include fever (104°F), headache, muscle pain, pain behind the eyes, joint pain, nausea, vomiting, rash, and swollen glands (Hadinegoro, 2012; Deen et al., 2006). In severe cases, symptoms such as acute abdominal pain, continuous vomiting, breathing difficulties, bleeding from the gums or nose, blood in vomit or stool, restlessness, excessive thirst, pale and cold skin, and weakness are commonly reported (Hadinegoro, 2012; Deen et al., 2006). This study addresses the existing gap in integrated research on the prevalence, clinical symptoms, molecular epidemiology, and seasonality of dengue in Bangladesh. The main aim of this study was to evaluate the existing data on the molecular epidemiology and clinical characteristics of dengue outbreaks in Bangladesh.

## Methods

### Definition

The epidemiology of the dengue virus in this study was defined as the distribution and determinants of outbreak in various populations, as well as the steps taken to reduce the health effects on those populations (Hadinegoro, 2012; WHO, 2024; Deen et al., 2006). Clinical symptoms included both the signs and symptoms observed during and after the confirmation of the dengue infection. Transmission was defined as the spread of the dengue virus from infected humans to healthy humans through mosquito vectors (Sharif et al., 2023a).

This study included previous studies that provided data on the epidemiology, clinical symptoms, genetic diversity, and seasonality of the dengue virus. The reported cases were confirmed using NS1 and/or IgM/IgG tests and/or RT-PCR, with molecular sequencing confirming dengue positivity (Hadinegoro, 2012; WHO, 2024; Deen et al., 2006). This study was conducted in accordance with the standards of the Preferred Reporting Items for Systematic Review and Meta-Analysis (PRISMA) Statement and Cochrane Collaboration guidelines (Moher et al., 2015).

### Study design

This study was conducted in accordance with the principles of systematic reviews, following the guidelines outlined in the Cochrane Handbook and the guidance document provided by the Center for Reviews and Dissemination (CRD) at York University, United Kingdom (Higgins and Green, 2020; Zeng et al., 2015). The study followed several key steps: identifying clear objectives, selecting data sources, developing search strategies, reviewing research articles, collecting and selecting data, minimizing bias, analyzing data, and summarizing the findings (Moher et al., 2015). This study included data and findings from original epidemiological studies, clinical and case studies, outbreak investigations, genotypic surveillance studies, and online databases. As strict assessment parameters were not available for these studies, the quality of the selected articles was evaluated based on the reports provided by their authors (Sharif et al., 2023a; Higgins and Green, 2020; Zeng et al., 2015; Sharif et al., 2021).

### Data sources, search strategy, and selection criteria

Literature and data were collected from published original articles in databases such as MEDLINE (via PubMed), The New England Journal of Medicine (NEJM), Web of Science, EMBASE, Scopus, African Journals Online (AJOL), and The Lancet. Eligible articles and scientific studies published before 01 March 2024 were included in this study, with data extracted from sources written in the English language. A significant number of search terms included: “Dengue, Dengue virus, DENV, DENV-1, DENV-2, DENV-3, DENV-4, Bangladesh, Dengue outbreaks, Epidemiology of dengue, Epidemiology of dengue virus, Clinical features of dengue infection, Sign and Symptoms of dengue, Clinical characteristics of dengue, Cases of dengue, Prevalence, Molecular epidemiology, serotyping, genotyping, Transmission of dengue, Transmission of dengue virus, *Aedes* spp., Mosquito, mosquito-borne, vector-borne, arbovirus” and various combination of these terms. A single search was conducted for every term across different websites and databases.

We searched for data on dengue outbreaks across various databases, including the World Health Organization (WHO), the Centers for Disease Control and Prevention (CDC, United States), Epicenter, ProMed, the European Centers for Disease Control and Prevention (ECDC), and the Directorate General of Health Services (DGHS, Bangladesh). Daily and yearly updates on dengue outbreaks were monitored from these data sources. Additionally, preprint platforms such as SSRN, medRxiv, bioRxiv, and AAS Open Research were searched, though only data from peer-reviewed journals were included.

We also manually reviewed the first 10 pages of search results from Google Scholar to gather relevant information. Our focus was on the molecular epidemiology of dengue, covering areas such as prevalence, incidence, transmission dynamics, case reports, clinical history, genotypic variation, case fatality rate, and distribution of serotypes. We also included data on seasonality and the spatial distribution of dengue cases in non-endemic regions.

After conducting searches across the above-mentioned websites and databases, potential articles and information were selected by removing irrelevant data and conducting a thorough screening. Articles containing data on specific and relevant matters were selected, covering all districts, ethnicities, ages, sexes, seasonality, and clinical features. Studies focused on modeling and prediction, review articles, and meta-analysis, and those unrelated to the objectives of this study were excluded.

The quality of the selected articles was further evaluated by identifying and removing duplicates, as well as excluding letters to the editor, correspondence, or comments. The seasonal exclusion criteria were applied to studies with seasonality and environmental data. Additionally, we included only studies that provided data on specific serotypes and genotypes.

The risk of bias was assessed using the Systematic Review Center for Laboratory Animal Experimentation (SYRCLE) assessment tool and the JBI critical appraisal checklist for studies reporting prevalence data (Moher et al., 2015; Higgins and Green, 2020; Zeng et al., 2015). The SYRCLE scale consists of 10 parameters to measure biases in studies, including attrition bias, reporting bias, detection bias, performance bias, selection bias, and other potential biases. Bias for each parameter was calculated using outcomes of *yes*, *no*, and *unclear*, corresponding to low, high, and undefined bias, respectively (Sharif et al., 2023a; Higgins and Green, 2020; Sharif et al., 2021). In the JBI tool, nine parameters were used to evaluate the studies, with each parameter evaluated using the outcomes *yes*, *no*, *unclear*, or not applicable.

### Case definition

According to the WHO, a dengue case is defined differently depending on the situation. A *surveillance case* is defined as “A person who lived in, or traveled to, a dengue-endemic area with the onset of fever and two or more of the following: nausea/vomiting, rashes, aches and pains, positive tourniquet test, leukopenia, or any warning sign.” A *confirmed dengue case* is defined as positive in polymerase chain reaction (PCR), virus culture, or positive IgM in a single sample, IgM seroconversion in paired sera, IgG seroconversion in paired sera, or a fourfold IgG titer increase in paired sera, or detection of viral antigen NS1+ in a single serum sample (WHO, 2024). According to the national guidelines for the clinical management of dengue syndrome in Bangladesh, depending on the time of testing after the onset of symptoms, a positive result in any of the following tests—NS1 antigen, IgM /IgG test (MAC ELISA or Rapid ICT), RT-PCR, or virus isolation—can be considered diagnostic for dengue.

### Protocol

This study followed the Preferred Reporting Items for Systematic Review and Meta-Analyses (PRISMA) guidelines (Moher et al., 2015).

### Statistical analysis

The total number of dengue cases and fatalities was calculated by summing the reported confirmed cases from the selected articles. The case fatality rate, defined as the proportion of dengue cases that result in death within a specified time period, was also determined. Pooled statistical analyses were conducted using Statistical Analysis System version 9.4 (North Carolina, United States).

## Results

### Studies included

We found 816 research articles on the epidemiology, genetic diversity, transmission, clinical characteristics, and seasonality of the dengue virus in Bangladesh using the previously mentioned search terms. Initially, 235 articles were selected for full-text analysis. The remaining 581 articles were excluded as they were reviews, mini-reviews, correspondence, or letters to the editor and did not meet inclusion criteria.

**Figure 1:**
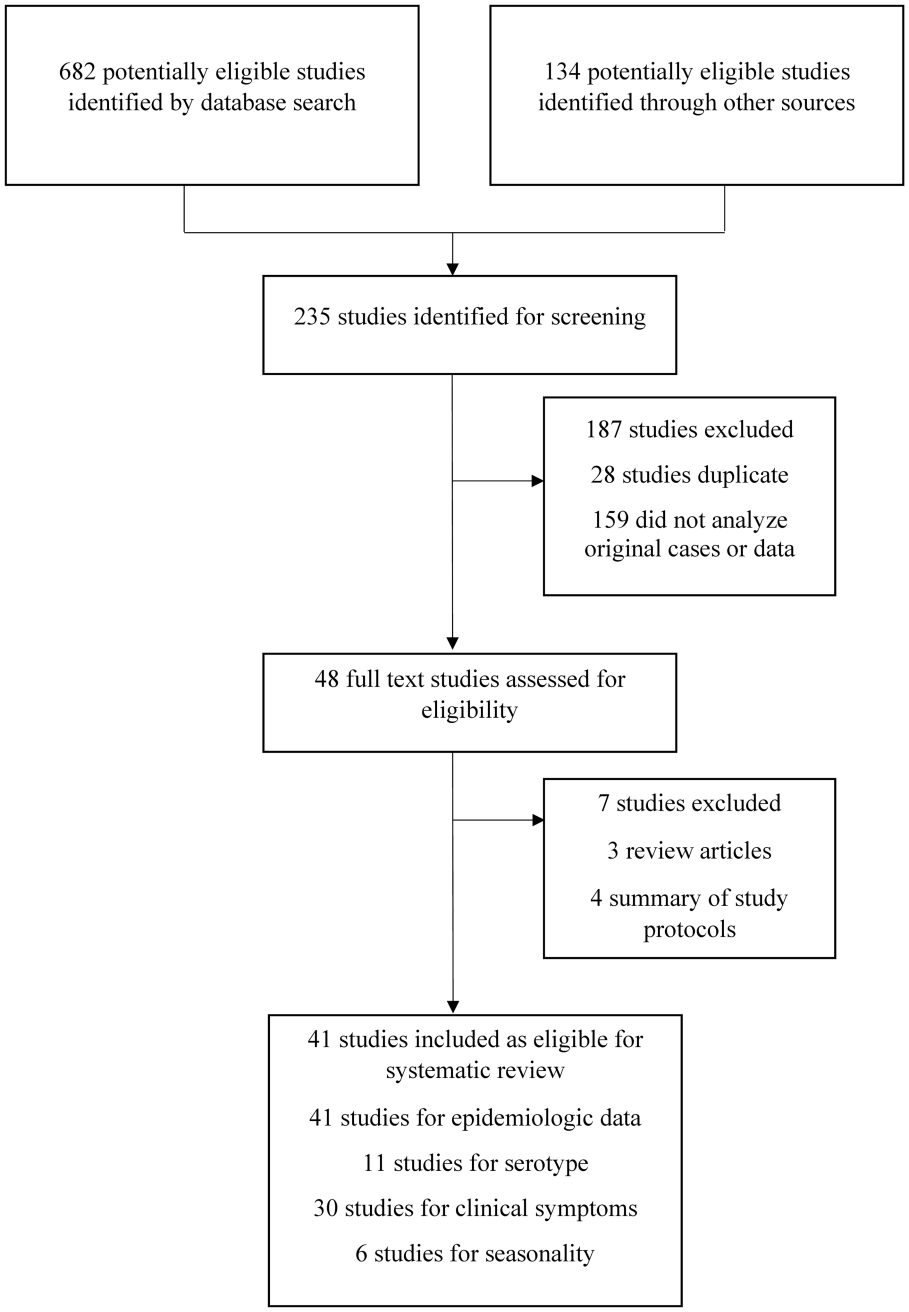
Selection and screening procedures of original studies. The excluded articles were irrelevant, duplicate, systematic reviews other than original studies and failed to meet inclusion criteria.

After a critical evaluation of the full texts, only 48 studies (20.5%, 48 of 235) were selected for further analysis. Based on the inclusion and exclusion criteria, 41 of 48 manuscripts (85.4%) were further analyzed. Among these 41 manuscripts, we extracted epidemiological data from all 41, clinical symptoms from 30 articles, genotypic diversity from 11 articles, and seasonality data from 6 articles (Figure 1). We also included data from two websites, including DGHS (Bangladesh) and Nextstrain.

### Epidemiological features of dengue outbreaks

**Figure 2:**
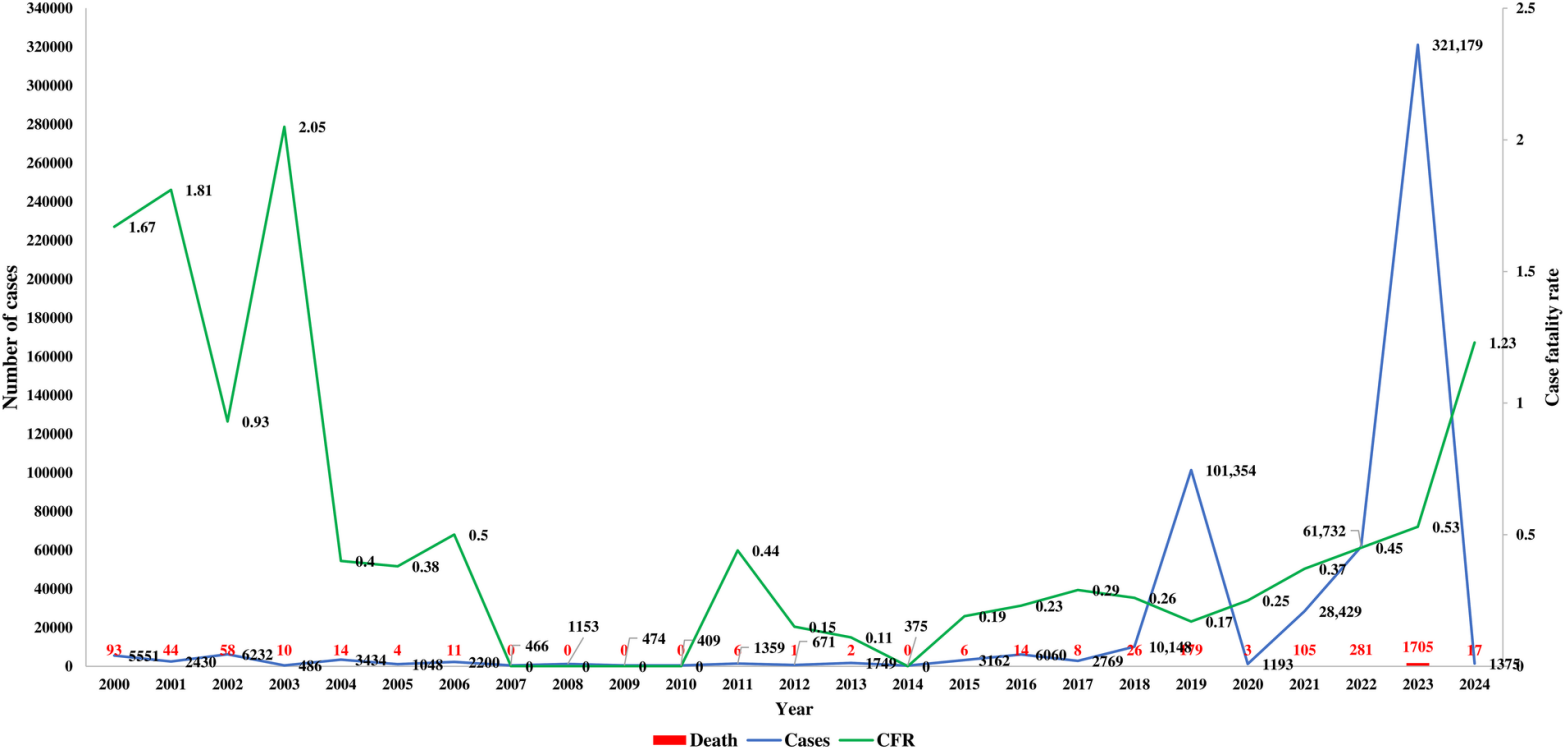
Trends of cases, fatalities, and CFR among the residents in Bangladesh.

Published articles and databases were assessed to examine the temporal and spatial trends of dengue in Bangladesh. The first major outbreak, with over 5,000 confirmed cases and 93 fatalities reported in 2000. From 2000 to 2009, approximately 24,000 cases were documented, resulting in 234 deaths. During this period, dengue incidence was concentrated in the hotspot of Dhaka (Figure 2). However, the source of the first case in Dhaka could not be confirmed, and the history of dengue transmission before 2000 was not thoroughly investigated in Bangladesh. The case fatality rate was highest from 2000 to 2003, with no fatality reported from 2007 to 2010.

From 2010 to 2019, 130,000 cases and 250 deaths were recorded, with over 100,000 cases reported for the first time in 2019 (WHO Dengue fact sheet, 2024; Hadinegoro, 2012). Moreover, 179 deaths due to dengue infection were reported in 2019 alone. The massive outbreak in 2023 saw confirmed cases surpass 415,000, with 2,200 deaths reported from dengue infection over the four-year period from 2020 to 2024. After the onset of COVID-19, dengue cases were significantly underreported in 2021 and 2022 (Figure 2). The 2022 dengue outbreak, continuing into 2024, is particularly alarming. The highest number of cases (56.8%, 321,179 of 565,438) and fatalities (65.9%, 1,705 of 2,587) were reported from January 2023 to December 2023.

The majority of the studies included patients from all age groups, with a male-to-female case ratio of approximately 2:1 (Table 1). Cases of dengue were most prevalent among individuals aged 19–29 years (31%), followed by 0–18 years (26%), 40–59 years (19.2%), 30–39 years (15.8%), 60–79 years (7.6%), and > 80 years However, fatalities were more frequent among women (70%). All of the studies (100%) confirmed cases using the NS1Ag test, followed by anti-dengue IgM and IgG antibody testing. Molecular sequencing and confirmation by RT-PCR were found in fewer than 10% of studies (Table 1) (WHO, 2024; Deen et al., 2006; Prattay et al., 2022; Muraduzzaman et al., 2018; Islam et al., 2022b; Ahmed et al., 2016; Islam et al., 2019; Islam et al., 2022a; Khan et al., 2021; Hasan et al., 2021a; Yang et al., 2023; Rafi et al., 2020; Shultana et al., 2019; Mahmood et al., 2021; Yesmin et al., 2023b; Rahim et al., 2023; Islam et al., 2006; Aziz et al., 2002; Sultana et al., 2020; Zeng et al., 2015; Sharif et al., 2021; Titir et al., 2021; Rahman et al., 2002; Rouf et al., 2020; Siddiqua et al., 2018; Yasmin et al., 2020; Nahar et al., 2021; Islam et al., 2021; Parvin et al., 2022). The epidemiological trends indicate a rapid increase in the incidence and fatalities from dengue in recent years in Bangladesh.

**Table 1:** Prevalence and epidemiology of dengue outbreaks in Bangladesh during 2000–2024.

Study	Region/Time	Number of participants	Age	Sex ratio (Male: Female)	Prevalence	Diagnosis method
Prattay et al. (2022)	Dhaka, 2019	336	All age	2.2:1	Only dengue-positive patients were included	NS1Ag and anti-dengue IgM and IgG antibody test
Muraduzzaman et al. (2018)	Dhaka, Chattogram, Khulna, 2013–2016	1,380	All age	N/A	42% (2013)	ELISA, RT-PCR
					21% (2014)	
					16% (2015)	
					13% (2016)	
Islam et al. (2022b)	Tangail, 2019	123	5 days to 17 years	62:38	Only dengue-positive patients were included	NS1Ag and anti-dengue IgM antibody test
Ahmed et al. (2016)	Dhaka, 2004	198	All age	4:1	Only dengue-positive patients were included	NS1Ag and anti-dengue IgM antibody test
Islam et al. (2019)	Dhaka, 2018	82	6 months to 15 years	1:1	Only dengue-positive patients were included	NS1Ag and anti-dengue IgM antibody test
Islam et al. (2022a)	Dhaka, 2019–2020	478	All age	1.23:1	Only dengue-positive patients were included	NS1Ag test
Khan et al. (2021)	Dhaka, 2019	190	<15 years	1.22:1	Only dengue-positive patients were included	NS1Ag and anti-dengue IgM antibody test
Hasan et al. (2021a)	Dhaka, 2019	747	All age	63:37	74% (553)	NS1Ag and anti-dengue IgM antibody test
Yang et al. (2023)	Dhaka, 2019	1,090	All age	60:40	Only dengue-positive patients were included	NS1Ag test
Rafi et al. (2020)	Bogra, 2019	319	All age	70:30	Only dengue-positive patients were included	NS1Ag and anti-dengue IgM antibody test
Shultana et al. (2019)	Dhaka, 2018	89	< 15 years	1.2:1	Only dengue-positive patients were included	NS1Ag and anti-dengue IgM antibody test
Mahmood et al. (2021)	Dhaka, 2019	542	All age	60:40	Only dengue-positive patients were included	NS1Ag and anti-dengue IgM antibody test
Yesmin et al. (2023b)	Dhaka, 2019	369	>18 years	60:40	Only dengue-positive patients were included	NS1Ag and anti-dengue IgM antibody test
Rahim et al. (2023)	Dhaka, 2018–2022	3,759	All age	60:40	839 (22.3%)	NS1Ag test, RT-PCR
Islam et al. (2006)	Dhaka, 2002	200	All age	2.7:1	100 (50%)	ELISA, RT-PCR
Aziz et al. (2002)	Dhaka, 2004–2005	45	All age	2:1	Only dengue-positive patients were included	ELISA, RT-PCR
Sultana et al. (2020)	Dhaka, 2018	316	N/A	N/A	Only dengue-positive patients were included	NS1Ag and anti-dengue IgM antibody test
Hossain et al. (2003)	Dhaka, 1996–1997	409	All age	55:45	Only dengue-positive patients were included	Widal test, anti-dengue IgM, and IgG antibody test
Amin et al. (2022)	Dhaka, 2018	297	All age	60:40	Only dengue-positive patients were included	NS1Ag and anti-dengue IgM antibody test
Kabir et al. (2020)	Noakhali, 2020	52	All age	80:20	Only dengue-positive patients were included	N/A
Titir et al. (2021)	Dhaka, Rangpur, Mymensingh, Sylhet, Chattogram, Barisal, Khulna, Jessore, Kustia, 2019	179	All age	61.5:38.5	162 (90.5%)	NS1Ag and anti-dengue IgM antibody test
Rahman et al. (2002)	Dhaka, 2000	336	All age	N/A	176 (73.3%)	ELISA, RT-PCR
Rouf et al. (2020)	Dhaka, 2018–2019	343	18 years and above	1.2:1	62 (18.1%)	NS1Ag and anti-dengue IgM antibody test, RT-PCR
Siddiqua et al. (2018)	Dhaka, 2015–2017	3,201	All age	N/A	1,037 (32.4%)	NS1Ag test, RT-PCR
Yasmin et al. (2020)	Dhaka, 2019	100	2 months–14 years	58:42	Only dengue-positive patients were included	NS1Ag and anti-dengue IgM antibody test
Nahar et al. (2021)	Dhaka, 2019	213	All age	1.7:1	Only dengue-positive patients were included	NS1Ag and anti-dengue IgM and IgG antibody test
Islam et al. (2021)	Dhaka, 2019	220	All age	53.6:46.4	Only dengue-positive patients were included	NS1Ag and anti-dengue IgM antibody test
Parvin et al. (2022)	Dhaka, 2019	100	>12 years	1.04:1	Only dengue-positive patients were included	NS1Ag and anti-dengue IgM and IgG antibody test
Mobarak et al. (2018)	Dhaka, 2016	56	1 year to 18 years	55:45	Only dengue-positive patients were included	NS1Ag and anti-dengue IgM and IgG antibody test
Sultana et al. (2019)	Dhaka, 2018	899	All age	69.3:30.7	350 (38.93%)	NS1Ag and anti-dengue IgM antibody test
Rahman et al. (2019)	Dhaka, 2019	70	>12 years	79:21	Only dengue-positive patients were included	NS1Ag and anti-dengue IgM antibody test
Pervin et al. (2017)	Dhaka, 2016	145	All age	62.5:37.5	40 (27.6%)	NS1Ag and anti-dengue IgM antibody test
Sultana et al. (2013)	Chattogram, 2009–2010	1,181	All age	N/A	533 (45.13%)	anti-dengue IgM and IgG antibody test
Datta et al. (2021)	Chattogram, 2019	192	< 12 years	59.4:40.6	Only dengue-positive patients were included	NS1Ag and anti-dengue IgM antibody test
Hasan et al. (2021b)	Dhaka, 2019	747	27 ± 31 y (range 3–85 y)	62.7:37.3	74%	NS1 antigen or anti-dengue immunoglobulin M (IgM).
Sharmin et al. (2013)	Dhaka (2008)	201	All	72.5:27.5	137 (68.2)	Anti-dengue immunoglobulin M (IgM) and IgG
Rahman et al. (2007)	Dhaka (2006)	225	36.86+/−17.60 years		156 (69.3)	Anti-dengue immunoglobulin M (IgM) and IgG
Salma et al. (2021)	Dhaka (2019)	4,200	All	N/A	All	Anti-dengue immunoglobulin M (IgM) and IgG
Yesmin et al. (2023a)	Dhaka, Tangail (2019)	208	<18 years	N/A	All	Anti-dengue immunoglobulin M (IgM) and IgG
Uddin et al. (2014)	Dhaka (2008–2010)	262	All	N/A	All	Anti-dengue immunoglobulin M (IgM) and IgG
Mutanabbi et al. (2022)	Dhaka (2019)	50	<12 years old	62:38	All	NS1 antigen or dengue IgM or IgG antibodies

### Spatial distribution of dengue in Bangladesh

The spatial distribution and transmission of dengue cases in Bangladesh have shifted significantly over the past two decades. The majority of the cases between 2000 and 2011 were indigenous to Dhaka, with 100% of cases in Khulna, Rajshahi, Mymensingh, Barisal, and Sylhet being transported from Dhaka. More than 500 transported cases were reported in Khulna, Barisal, Chattogram, Mymensingh, Sylhet and Rajshahi. Before the larger outbreak in 2019, dengue was reported in only 30% of regions in Bangladesh. However, from 2012 to 2023, the distribution of dengue cases rapidly expanded to all 64 districts (100% regions) (WHO Dengue fact sheet, 2024; Health bulletin on current Dengue situation published by DGHS, 2024; Parvin et al., 2022; Mobarak et al., 2018; Sultana et al., 2019; Rahman et al., 2019; Pervin et al., 2017; Sultana et al., 2013; Datta et al., 2021; Hasan et al., 2021b; Sharmin et al., 2013; Rahman et al., 2007; Salma et al., 2021; Yesmin et al., 2023a).

**Figure 3:**
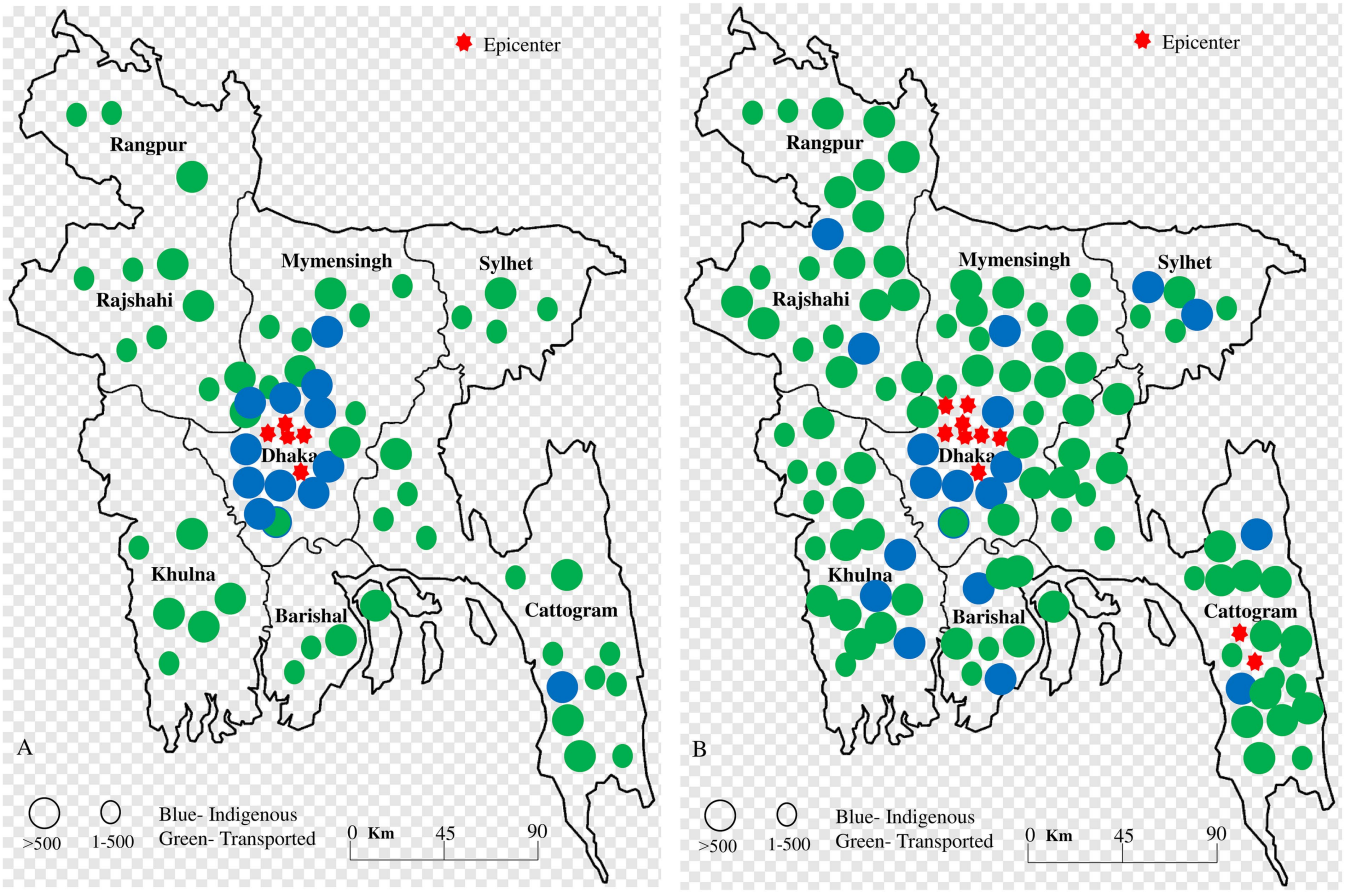
Spatial distribution of indigenous and transported cases (A) during 2000–2011 and (B) 2012–2023. The blue color indicated indigenous cases in specific regions, while the green color indicated transported cases. The red star indicated a hotspot of outbreaks. During 2000–2011, the majority of indigenous cases were confined to central regions in Bangladesh. However, from 2012 to 2023, the southern and southeastern regions became highly infected, and indigenous cases became more common throughout Bangladesh.

During the 2019 outbreak, the dengue virus spread to the remaining 70% of regions in Bangladesh, with Dhaka being the focal point for the 2019, 2021, 2022, and 2023 outbreaks (Figure 3). However, during the 2023 outbreak, indigenous cases were locally transmitted in regions such as Chattogram, Rajshahi, Sylhet, Khulna, and Barisal. In 2023, 53% of cases were reported outside Dhaka, with the Chattogram division accounting for 14.2% of cases and the Barisal division for 11%, making them new major hotspots of dengue transmission after 2023 (Yesmin et al., 2023a; Uddin et al., 2014; Mutanabbi et al., 2022; Pervin et al., 2003; Baskey et al., 2024; Roy et al., 2023; Rahim et al., 2021).

### Serotype and genetic diversity of dengue viruses in Bangladesh

**Figure 4:**
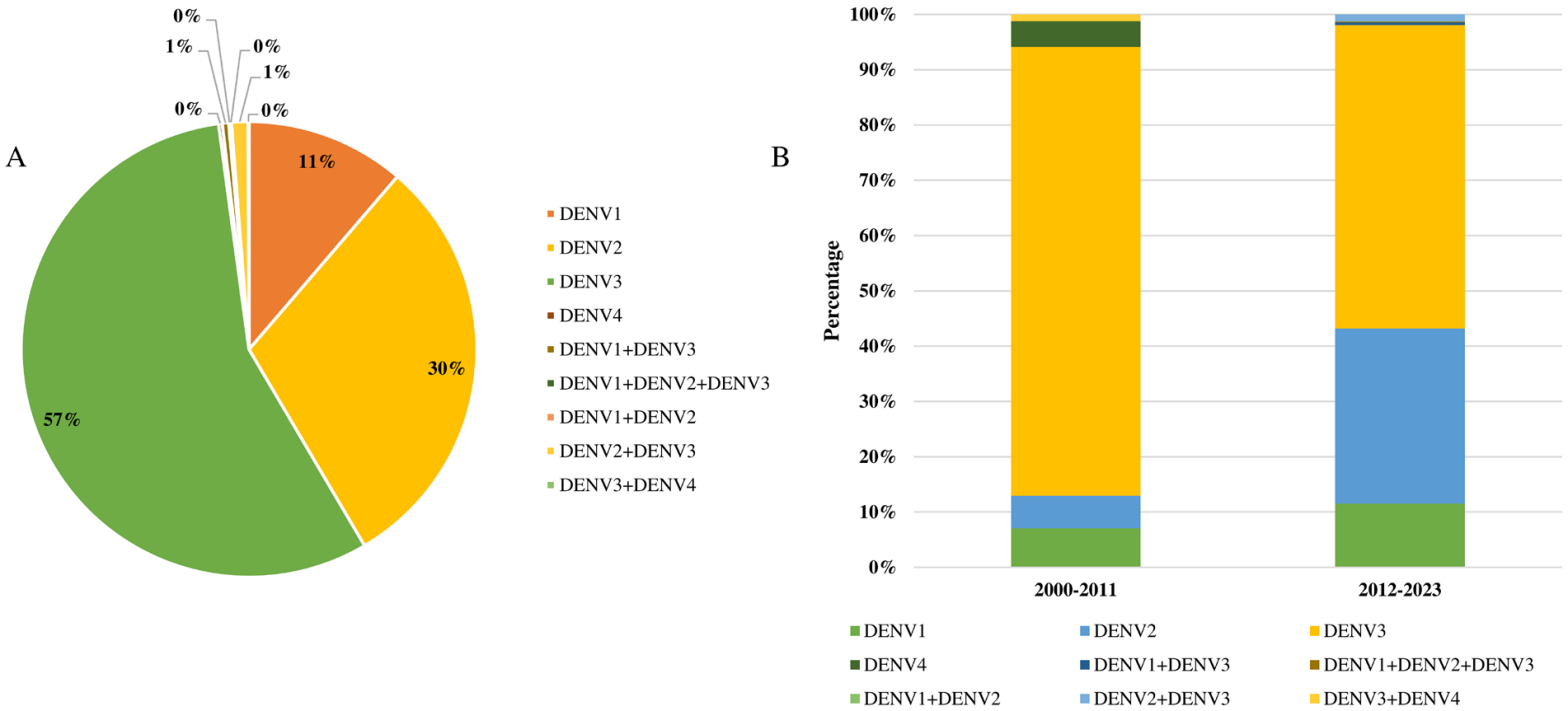
(A) Proportionate frequency of serotypes of dengue virus in Bangladesh during 2000–2024. (B) Temporal distributions of serotypes of dengue virus in Bangladesh.

Data on the serotype diversity and genotypic characteristics of the dengue virus in Bangladesh are significantly lacking. We identified 11 studies that examined dengue virus serotype diversity in Bangladesh from 2000 to 2024. The majority of studies (100%) on the genotyping of dengue viruses were conducted in Dhaka, with a total of 1,575 samples analyzed. Serotyping was conducted on 92.4% (1,456 of 1,575) of samples during the period from 2012 to 2023, compared to only 5.4% (85 of 1,575) between 2000 and 2011 (Figure 4). Among the four serotypes, DENV3 was the most prevalent (57%, 868 of 1,541), followed by DENV2 (30%, 466 of 1,541), DENV1 (11%, 174 of 1,541), and mixed DENV2 and DENV3 infections (1%, 18 of 1,541). The diversity of serotypes and the occurrence of mixed infections increased during 2012–2023 (Figure 4).

DENV4 (4 of 1,541) and mixed infections of DENV3 and DENV4 were only reported in Dhaka in 2000. Additionally, mixed infections of DENV1 and DENV3 (7 cases) and DENV1, DENV2, and DENV3 (2 cases) were reported in Dhaka in 2018. The genotypic diversity of dengue viruses in Bangladesh remains poorly studied. Among the genotypes, DENV3-I was the most prevalent (43 of 59), followed by DENV3-II (8 of 59), DENV3-III (2 of 59), and mixed genotype DENV3-I, III (6 of 59) (Supplementary Table S1). The cosmopolitan DENV2 genotype was reported in 2019, while the cosmopolitan DENV3 was identified in 2018 in Dhaka.

Phylogenomic analysis of dengue virus also confirmed the circulation of DENV2 and DENV3 genotypes (divergence 0.39) in Bangladesh. Furthermore, phylogenetic analysis of 12 isolates based on the E gene showed that 100% clustered with DENV3 and were closely related to isolates from Thailand, the Philippines, Indonesia, and Australia (Supplementary Figure S1). The sequence similarity of Bangladeshi DENV3-II strains was exceptionally higher (99.93%) than that of strains from India.

### Clinical characteristics of patients with dengue virus infection

We extracted clinical data from 30 articles, 29 of which were conducted before the 2023 outbreak. The majority of the study (>90%) found fever, headache, rash, vomiting, abdominal pain, and diarrhea as the most prevalent symptoms, followed by arthralgia, myalgia, retro-orbital pain, nausea, fatigue, fluid leakage, and back pain (reported in 70–89% of studies) (Table 2). Body aches, malaise, petechiae, hemorrhage, gum bleeding, nasal bleeding, and hematuria were less commonly reported symptoms (<50% of the studies).

**Table 2:** Clinical manifestations among persons infected with dengue virus in Bangladesh during 2000–2023.

Study	Dengue-positive	Symptoms, *N* (%)															
		Fever	Headache	Body ache	Retro-orbital pain	Arthralgia	Myalgia	Anorexia	Rash	Nausea	Vomiting	Abdominal pain	Back pain	Diarrhea	Fluid leakages	Hemorrhage	Gum bleeding
Prattay et al. (2022)	336	301 (98)	71 (21)	132 (39)	N/A	7 (2)	N/A	60 (18)	18 (5)	45 (13)	99 (29)	41 (12)	8 (2)	26 (8)	N/A	N/A	3 (1)
Islam et al. (2022b)	123	123 (100)	70 (57)	71 (58)	26 (21)	N/A	N/A	N/A	68 (55)	N/A	N/A	36 (29)	N/A	24 (19)	N/A	37 (30)	N/A
Ahmed et al. (2016)	198	196 (99)	189 (96)	N/A	N/A	N/A	N/A	13 (6)	N/A	29 (15)	57 (29)	33 (17)	N/A	17 (9)	174 (88)	31 (15)	41 (21)
Islam et al. (2019)	82	82 (100)	15 (18)	N/A	10 (12)	13 (16)	39 (48)	N/A	57 (69)	N/A	53 (65)	49 (60)	N/A	9 (11)	48 (58)	N/A	10 (12)
Islam et al. (2022a)	478	452 (95)	234 (49)	41 (9)	260 (54)	324 (68)	N/A	N/A	280 (58)	N/A	153 (32)	N/A	N/A	57 (12)	N/A	N/A	49 (10)
Khan et al. (2021)	190	190 (100)	126 (68)	N/A	63 (34)	75 (41)	N/A	N/A	52 (28)	N/A	152 (80)	122 (65)	66 (37)	81 (43)	42 (24)	N/A	N/A
Hasan et al. (2021a)	553	553 (100)	347 (63)	N/A	216 (39)	25 (4)	N/A	210 (38)	25 (4)	385 (70)	385 (69)	230 (42)	58 (10)	145 (26)	N/A	N/A	20 (7)
Yang et al. (2023)	1,090	1,034 (95)	901 (83)	N/A	N/A	516 (47)	691 (63)	N/A	277 (25)	N/A	837 (77)	631 (58)	651 (60)	N/A	272 (25)	159 (15)	N/A
Rafi et al. (2020)	319	295 (93)	232 (73)	N/A	150 (47)	N/A	228 (71)	N/A	50 (16)	N/A	109 (34)	95 (30)	N/A	138 (43)	38 (12)	N/A	25 (9)
Shultana et al. (2019)	89	89 (100)	11 (12)	N/A	1 (1)	12 (13)	12 (13)	N/A	43 (48)	33 (37)	33 (37)	21 (24)	12 (13)	8 (67)	10 (11)	23 (26)	1 (1)
Mahmood et al. (2021)	542	505 (93)	249 (46)	N/A	27 (5)	N/A	146 (27)	37 (7)	137 (25)	331 (61)	331 (61)	160 (29)	-	107 (20)	N/A	N/A	N/A
Yesmin et al. (2023b)	369	369 (100)	223 (60)	N/A	132 (36)	75 (20)	73 (20)	162 (44)	64 (17)	249 (67)	249 (67)	136 (37)	104 (28)	134 (36)	91 (24)	83 (22)	22 (6)
Rahim et al. (2023)	67	N/A	16 (24)	23 (34)	N/A	N/A	N/A	12 (18)	9 (13)	36 (53)	36 (54)	37 (55)	N/A	6 (9)	10 (15)	11 (16)	N/A
Islam et al. (2006)	100	100 (100)	96 (96)	N/A	N/A	91 (91)	N/A	N/A	28 (28)	N/A	93 (93)	83 (83)	N/A	N/A	N/A	7 (7)	41 (41)
Aziz et al. (2002)	45	45 (100)	40 (89)	40 (89)	N/A	N/A	N/A	38 (84)	10 (22)	N/A	41 (91)	N/A	N/A	N/A	9 (20)	5 (11)	N/A
Amin et al. (2022)	297	287 (96.6)	269 (91)	79 (27)	151 (51)	87 (28)	79 (26)	239 (80)	98 (33)	227 (76)	227 (76)	100 (34)	218 (73)	115 (39)	43 (14)	10 (3)	6 (2)
Kabir et al. (2020)	52	52 (100)	34 (65)	N/A	N/A	N/A	N/A	N/A	8 (15)	23 (44)	23 (44)	7 (13)	N/A	N/A	N/A	N/A	N/A
Rahman et al. (2002)	176	176 (100)	160 (91)	N/A	N/A	150 (85)	150 (85)	N/A	97 (55)	N/A	113 (64)	N/A	N/A	N/A	81 (46)	21 (12)	20 (11)
Rouf et al. (2020)	25	15 (60)	25 (100)	25 (100)	N/A	N/A	N/A	6 (24)	N/A	6 (24)	7 (28)	5 (20)	N/A	4 (16)	N/A	N/A	N/A
Siddiqua et al. (2018)	100	100 (100)	60 (60)	60 (60)	N/A	N/A	N/A	N/A	N/A	66 (66)	66 (66)	66 (66)	N/A	N/A	N/A	36 (36)	22 (22)
Yasmin et al. (2020)	213	213 (100)	24 (11)	23 (11)	23 (11)	23 (11)	N/A	N/A	N/A	51 (24)	51 (24)	46 (22)	N/A	28 (13)	N/A	104 (49)	N/A
Islam et al. (2021)	220	202 (92)	106 (48)	N/A	31 (14)	N/A	43 (19)	N/A	32 (14)	N/A	110 (50)	108 (49)	N/A	121 (55)	27 (12)	114 (52)	N/A
Parvin et al. (2022)	100	63 (63)	69 (69)	N/A	40 (40)	27 (27)	42 (42)	N/A	14 (14)	N/A	70 (70)	77 (77)	N/A	N/A	68 (68)	39 (39)	8 (8)
Mobarak et al. (2018)	56	56 (100)	38 (68)	N/A	41 (73)	35 (62)	43 (76)	N/A	31 (55)	N/A	29 (52)	26 (46)	N/A	N/A	31 (55)	N/A	N/A
Sultana et al. (2019)	350	350 (100)	214 (61)	N/A	71 (20)	81 (23)	154 (44)	N/A	21 (6)	N/A	N/A	N/A	11 (3)	18 (5)	N/A	N/A	N/A
Rahman et al. (2019)	70	64 (91)	62 (88)	N/A	36 (51)	45 (64)	N/A	59 (85)	12 (17)	49 (71)	46 (67)	19 (27)	47 (67)	N/A	3 (4)	N/A	N/A
Pervin et al. (2017)	40	40 (100)	10 (25)	N/A	20 (50)	35 (87)	5 (12)	11 (27)	5 (12)	11 (27)	11 (27)	12 (30)	N/A	10 (25)	11 (27)	N/A	N/A
Datta et al. (2021)	192	192 (100)	45 (23)	N/A	N/A	28 (14)	28 (14)	N/A	22 (11)	N/A	92 (48)	176 (92)	N/A	N/A	N/A	N/A	N/A
Sharif et al. (2024a)	47,854	(99)	(81)	(71)	N/A	(86)	(86)	N/A	(<10)	N/A	(62)	N/A	N/A	(65)	(65)	(74)	N/A

Approximately 6,788 cases with documented symptoms were reported across the 29 articles from dengue outbreaks between 2000 and 2022. Fever was the most prevalent symptom (90.51, 95% CI 85–100%), followed by headache (57.98, 95% CI 12–100%), vomiting (51.16, 95% CI 23–91%), abdominal pain (34.12, 95% CI 12–85%), myalgia (25.53, 95% CI 13–85%), arthralgia (24.29, 95% CI 8–91%), nausea (22.70, 95% CI 13–76%), rash (21.48, 95% CI 7–70%), fatigue (20.14, 95% CI 8–80%), retro-orbital pain (19.12, 95% CI 3–51%), back pain (17.31, 95% CI 3–73%), and diarrhea (15.44, 95% CI 7–66%). Furthermore, among the severe symptoms, fluid leakage (14.1, 95% CI 5–88%) was the most prevalent, followed by hemorrhage (11, 95% CI 3–51%), gum bleeding (10, 95% CI 1–41%), and hematuria (8, 95% CI 1–16%).

During the 2023 outbreak, changes in the frequency of symptoms were found. Fever was the most frequent symptom (99%), followed by myalgia (86%), anorexia (86%), fatigue (86%), headache (81%), malaise (81%), hemorrhage (74%), body ache (71%), diarrhea (65%), and vomiting (65%). The rash was found in fewer than 10% of patients during the 2023 outbreak (Table 2). Mild to moderate symptoms were reported in 85% of patients, with severe outcomes in 12% and death in fewer than 1% of cases.

### Seasonality of dengue outbreaks in Bangladesh

**Figure 5:**
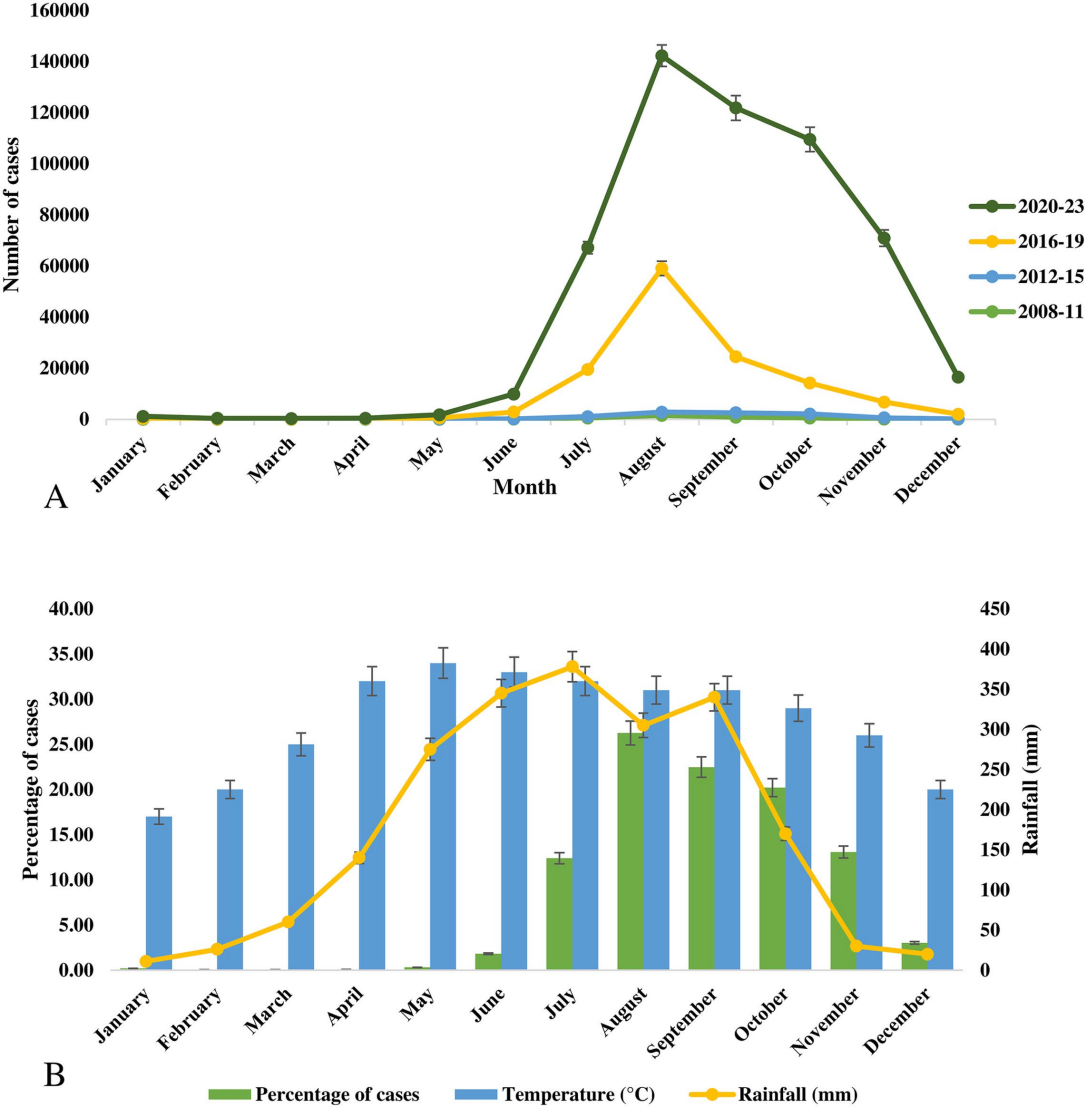
(A) Monthly distribution of cases of dengue outbreaks during 2008–2023 in Bangladesh. (B) Frequency distribution of cases of dengue virus, average temperature and rainfall in Bangladesh.

Seasonal changes in dengue cases and outbreaks have been documented in Bangladesh. While the existing literature and databases offer limited information on the seasonality of dengue, we compiled all available data. Cases and fatalities increased sharply after June in Bangladesh, with a peak in outbreaks occurring between July and November. Among the documented 541,751 cases documented from 2008 to 2023, the highest frequency was recorded in August (26.3%), followed by September (22.5%), October (20.2%), November (13.08%), and July (12.4%) (Figure 5). The first 6 months of the year (January to June) contributed to less than 3% of total dengue cases in Bangladesh.

The seasonal pattern of the 2023 outbreak, which was the largest in Bangladesh’s history, was similar to previous outbreaks with minor variations. In 2023, the highest number of cases was reported in September (79,598 cases), followed by August (71,976 cases), October (67,769 cases), July (43,854 cases), and November (40,716 cases). The seasonal changes in the vector density have a strong impact on these outbreaks. Although Bangladesh lacks a strong vector surveillance program, recent studies from 2021 to 2023 have shown a high abundance of *Aedes aegypti* mosquitoes in Dhaka (Haque et al., 2023; Sim et al., 2020). Additionally, the rapid spread of dengue from 2019 to 2024 in non-endemic regions was driven not only by imported cases but also by indigenous transmissions. The vector has now spread to most districts in Bangladesh and has adapted to local climatic conditions.

Average temperatures in Bangladesh vary from 17°C (January) to 34°C (May). In the peak dengue months, the average temperature was 32°C in July, 31°C in August and September, 29°C in October, and 26°C in November. The highest average rainfall was recorded in July (378 mm), followed by June (345 mm), September (340 mm), August (305 mm), and May (275 mm) (Figure 5).

### Future perspectives of dengue outbreaks in Bangladesh

The 2023 outbreak was the largest on record, with 400,000 confirmed cases, followed by the 2019 outbreak with 120,000 confirmed cases in Bangladesh. Massive transmission of dengue cases occurred in non-endemic regions during the 2019 and 2023 outbreaks, with all 64 districts affected. In 2023, 70% of cases were reported outside Dhaka, the traditional focal point. Without specific treatments or an approved vaccine, dengue poses a major health threat in Bangladesh.

The introduction of an effective dengue vaccine could mitigate this health risk, as evidenced by the recent randomized trial of the TV005 tetravalent vaccine in Bangladesh. Integrated vector management (IVM), along with community engagement and the introduction of innovative approaches such as *Wolbachia*-mediated biocontrol, could help reduce dengue transmission. Although Bangladesh’s national dengue surveillance system has improved, active tracing and real-time monitoring are needed to assess the true burden of the disease.

Factors such as mixed and cross-infections, the circulation of DENV1 and DENV4 in neighboring countries, rapid communication, high population density, lack of awareness, insufficient genotypic characterization, a weak national vector management policy, changing climate conditions, and evolving vector characteristics will likely contribute to future major dengue outbreaks, potentially infecting millions of people in Bangladesh in the future.

## Discussion

Dengue outbreaks have become a major health threat in Bangladesh (Health bulletin on current Dengue situation published by DGHS, 2024; Siddiqua et al., 2018; Nahar et al., 2021; Parvin et al., 2022; Mobarak et al., 2018; Sultana et al., 2019; Rahman et al., 2019; Pervin et al., 2017; Yesmin et al., 2023a; Rahim et al., 2021). The magnitude of recent dengue outbreaks has increased significantly, with countrywide transmission in 2019 and 2023 infecting approximately half a million (Health bulletin on current Dengue situation published by DGHS, 2024; Zeng et al., 2015; Sharif et al., 2021; Titir et al., 2021; Rahman et al., 2002; Rouf et al., 2020; Siddiqua et al., 2018; Yasmin et al., 2020; Nahar et al., 2021; Mutanabbi et al., 2022; Pervin et al., 2003; Baskey et al., 2024; Roy et al., 2023; Rahim et al., 2021). Integrated studies on the prevalence, molecular epidemiology, clinical characteristics, and seasonality are crucial to provide a more accurate picture of dengue outbreaks in Bangladesh. The 2019 and 2023 outbreaks accounted for 78% of all dengue cases in the country. Our analysis reveals not only an increase in cases and fatalities but also a significant geographic expansion of cases into previously non-endemic regions.

The sharp rise in cases and fatalities during the 2019 and 2023 outbreaks is largely associated with the rapid increase in vector mosquitoes and the wide distribution of their habitats, along with the evolving genotypes of the dengue viruses. The frequently evolving genotypes of DENV may be contributing to this rapid spread, and further studies are needed to explore this connection.

These findings are similar to those reported by previous studies in Bangladesh (Rahim et al., 2021; Ahmad et al., 2020; Sharif et al., 2024a). However, the rapid expansion of dengue across the country contrasts with trends observed in nearby countries and other dengue-endemic regions globally. The reported number of dengue cases from 2020 to 2022 was lower during the COVID-19 pandemic (Bhowmik et al., 2023; Hasan et al., 2019; Sharif et al., 2023b). This low number was primarily due to underreporting. Additionally, strict lockdowns and reduced intercity travel, particularly in Dhaka, may have contributed to the reduced number of cases.

During the 2019 outbreak and afterward, *Aedes aegypti* and *Aedes albopictus* rapidly spread across Bangladesh. As a result, in the 2023 outbreak, the majority of the cases were documented outside the established hotspot of Dhaka (Health bulletin on current Dengue situation published by DGHS, 2024; Rahim et al., 2021; Ahmad et al., 2020). Moreover, cases in non-endemic regions were non-travel-related and indigenous, with transmission documented from the beginning to the end of the outbreak. These findings are alarming not only for Bangladesh but also for other dengue-endemic regions worldwide.

Among the various demographic characteristics reported in most data sources, age and sex were commonly mentioned. We found the majority of the cases (70%) between 2000 and 2024 occurred in men. However, the case fatality rate was significantly higher among women, with a women-to-men ratio of 3:2. Factors contributing to the higher death rate among females include extended time at home, delayed visits to healthcare providers, and the presence of anemia. Previous studies from Bangladesh, as well as from India, the Philippines, Indonesia, China, and Pakistan, have supported these findings (Khan et al., 2021; Hasan et al., 2021a; Yang et al., 2023; Rafi et al., 2020; Datta et al., 2021; Salma et al., 2021; Yesmin et al., 2023a; Uddin et al., 2014; Mutanabbi et al., 2022; Pervin et al., 2003; Baskey et al., 2024; Roy et al., 2023; Rahim et al., 2021; Ahmad et al., 2020; Sharif et al., 2024b).

Both the spatial and temporal distribution of dengue cases have shown a rapid increase in cases in recent times (2018–2024). While global data suggest some improvement in many endemic regions due to integrated management, the situation in Bangladesh has been markedly different (Sharif et al., 2024b). If the uncontrolled outbreak continues, millions of people could be infected, and many could die from dengue. The 2023 outbreak was the worst and longest in Bangladesh’s history.

However, many cases were likely underreported due to a lack of advanced surveillance and limited diagnostic facilities (Rahim et al., 2021). Additionally, the dengue outbreak spread to rural areas where most residents had limited access to healthcare and low awareness of the disease. Consequently, asymptomatic individuals and those with mild symptoms were not included in the official documentation, making the actual health burden of recent dengue outbreaks much higher than documented. These observations are supported by previous studies in Bangladesh (WHO Dengue fact sheet, 2024; Health bulletin on current Dengue situation published by DGHS, 2024; Datta et al., 2021; Hasan et al., 2021b; Sharmin et al., 2013; Rahman et al., 2007; Salma et al., 2021; Rahim et al., 2021; Ahmad et al., 2020).

The highest frequency of cases in the 2023 outbreak was recorded in the Dhaka division (18.3%, 58,971 of 321,179 cases), followed by Chattogram (13.8%, 44,435 of 321,179 cases), Barishal (11.8%, 38,049 of 321,179), and Khulna (10.8%, 34,722 of 321,179 cases). The southern regions (Khulna and Barishal divisions) and the southeastern region (Chattogram division) emerged as new hotspots for dengue transmission. In the southern areas, both *Aedes aegypti* and *Aedes albopictus* mosquitoes are prevalent, while in the southeastern region, only *Aedes albopictus* has been reported. These findings are supported by previous studies in Bangladesh (WHO, 2024; Deen et al., 2006; Prattay et al., 2022; Muraduzzaman et al., 2018; Islam et al., 2022b; Ahmed et al., 2016; Islam et al., 2019; Islam et al., 2022a; Khan et al., 2021; Hasan et al., 2021a; Yang et al., 2023; Datta et al., 2021; Hasan et al., 2021b; Sharmin et al., 2013; Rahman et al., 2007; Salma et al., 2021; Yesmin et al., 2023a; Rahim et al., 2021; Ahmad et al., 2020; Sharif et al., 2024a; Bhowmik et al., 2023; Hasan et al., 2019; Sharif et al., 2023b).

The rapid spread of dengue in non-endemic regions and the larger outbreaks with uncontrolled cases in Bangladesh have been driven by the higher density and wider distribution of vector mosquitoes. Several factors contribute significantly to this situation, including environmental conditions, climate change, human activities, population density, poor management and policies, changes in vector characteristics, and the mosquitoes’ adaptation capabilities (Rahim et al., 2021; Ahmad et al., 2020). Increased average temperature, seasonal rainfall, and flooding have notably supported the reproduction, survival, and spread of *Aedes* spp. in most regions of Bangladesh.

The majority of the study confirms a seasonal spike in cases of dengue in Bangladesh, with the highest frequency occurring between August and October, an increase in cases beginning in July and lasting through November (Ahmad et al., 2020). According to previous studies, we also found that the optimal temperature range for *Aedes* spp. reproduction and spread is between 23°C and 29°C, with higher rainfall further facilitating their growth. Similarly, during the period from August to November, the average temperature in Bangladesh ranged from 26°C to 31°C, further supporting the seasonal rise in dengue cases.

Serotype and genotype data highlight a significant research gap in Bangladesh. Among the isolates, DENV3 (57%) was the most common serotype, followed by DENV2 (30%) and DENV1 (11%). However, DENV4 has not been reported recently. Additionally, the prevalence of mixed infections among different serotypes, including DENV1-DENV3, was also significant (2–10%). This raises concerns about the high risk of secondary and post-secondary infections in individuals exposed to different serotypes, which could further complicate the situation. The risk is heightened by direct vector transmission and travelers’ cases from nearby endemic countries, such as India, which could exacerbate the spread of dengue in Bangladesh. These findings are supported by previous studies (Health bulletin on current Dengue situation published by DGHS, 2024; WHO, 2024; Baskey et al., 2024; Rahim et al., 2021; Ahmad et al., 2020).

Genotype DENV3-I was the most prevalent (73%), followed by DENV3-II (13%). These genotypes show high similarity with isolates from India, the Philippines, Indonesia, and Australia (Hadinegoro, 2012). A recent study from Northern West Bengal, India, reported the prevalence of DENV1 and DENV3 of genotype III (Roy et al., 2023). Furthermore, a study in Kolkata found that the dominant serotypes of DENV evolved from DENV3 in 2015 to DENV1 in 2016 and DENV2 from 2017 to 2019 (Baskey et al., 2024). These studies support the idea that the importation of DENV from nearby countries could significantly affect the present dengue situation in Bangladesh. Our findings also suggest that studies on the diversity of genotypes in Bangladesh are still rare and require further attention.

Changes in the symptoms experienced by patients with dengue virus infection have been noted in existing studies. The number of patients presenting with critical health conditions has risen along with the increase in dengue cases from 2019 to 2023. Notably, there has been an increase in symptoms such as myalgia, anorexia, fatigue, headache, malaise, and hemorrhage, while the frequency of rashes has decreased. One possible explanation for these changing symptoms is the involvement of antibody-dependent enhancement (ADE). Secondary infections with different dengue serotypes can trigger immunoglobulin G (IgG)-mediated ADE, which may lead to greater disease severity in affected individuals. This theory aligns with recent studies conducted in Bangladesh (Sharif et al., 2021; Titir et al., 2021; Rahman et al., 2002; Rouf et al., 2020; Siddiqua et al., 2018; Yasmin et al., 2020; Nahar et al., 2021; Islam et al., 2021; Parvin et al., 2022; Mobarak et al., 2018; Sultana et al., 2019; Rahman et al., 2019; Pervin et al., 2017; Sultana et al., 2013; Datta et al., 2021; Hasan et al., 2021b; Sharmin et al., 2013; Rahman et al., 2007; Salma et al., 2021; Rahim et al., 2021; Ahmad et al., 2020).

Dengue virus-specific antivirals are currently unavailable, and dengue vaccines are not approved for use in Bangladesh. The efficacy of existing dengue vaccines is moderate to low and recommended only for those who have been previously infected. The absence of targeted treatments or vaccine-mediated prevention elevates the risk of larger outbreaks. While integrated vector management has been successful in reducing the burden in many countries, Bangladesh faces significant gaps in this regard.

Key contributing factors to the outbreaks include the lack of active surveillance in rural areas, limited public awareness of vector control, gaps in integrated and nationwide management, and the absence of a clear roadmap for vector control. Additionally, the adaptability of vectors, rapid urbanization, deforestation, climate change, rising temperatures, prolonged monsoon seasons, and increased movement and transportation all play vital roles in fueling larger dengue outbreaks in Bangladesh.

This study has a number of limitations. Genomic data needed more in-depth analysis to predict mutations and evolutionary changes that could be linked to altered epidemiological characteristics. Additionally, since this study focuses on the dengue virus, detailed information on vector characterization could not be included. However, the main strength of this study lies in its comprehensive analysis, which incorporates the most up-to-date data and removes biases. Furthermore, it addresses a wide range of epidemiological aspects related to dengue outbreaks in Bangladesh.

## Conclusion

This study concludes that dengue has recently become a major health burden in Bangladesh, affecting nearly 0.5 million people. Indigenous cases in non-endemic regions, particularly in the southern and southeastern parts of the country, have become more common than in the traditional hotspot of Dhaka since the 2019 outbreak. Serotypes DENV3 (57%) and DENV2 (30%) accounted for approximately 90% of characterized isolates in Bangladesh. The study highlights a significant gap in the genotypic and serotype characterization of the dengue virus in the country. Additionally, the lack of timely and effective policies for vector control has contributed to the rising number of cases. This study provides a valuable integrated dataset and serves as a guideline for future research, policymakers, and the scientific community.

## Data Availability

The original contributions presented in the study are included in the article/Supplementary material, further inquiries can be directed to the corresponding authors.
