# Supplementary material for: Evolving epidemiology, clinical features, and genotyping of dengue outbreaks in Bangladesh, 2000–2024: a systematic review
Source: Front Microbiol. 2024 Oct 30;15:1481418. doi: 10.3389/fmicb.2024.1481418 (PMC11557403; doi:10.3389/fmicb.2024.1481418)
Supplement: Supplementary file 1 [file Table_1.DOCX]

**Supplementary Table i.** Diversity and trends of dengue virus serotypes/genotypes during 2000-2024 in Bangladesh.

| **Study** | **Region/Time** | **Number of samples typed** | **Serotype (N)** | | | | | **Genotype (N)** |
| --- | --- | --- | --- | --- | --- | --- | --- | --- |
|  |  |  | **DENV1** | **DENV2** | **DENV3** | **DENV4** | **Mixed** |  |
| Muraduzzaman et al, (2018)^5^ | Dhaka, Chattogram, Khulna, 2013-2016 | 141 | 47 | 94 | 0 | 0 | N/A | 0 |
| Rahim et al, (2023)^17^ | Dhaka,  2018-2022 | 495 | 40 | 52 | 403 | 0 | N/A | 0 |
| Islam et al, (2006)^18^ | Dhaka, 2002 | 8 | 0 | 0 | 8 | 0 | N/A | DENV3-II (8) |
| Aziz et al, (2002)^19^ | Dhaka,  2000 | 19 | 0 | 2 | 14 | 0 | DENV2+DENV3 (2), DENV3+DENV4 (1) | 0 |
| Titir et al, (2021)^29^ | Dhaka, Rangpur, Mymensingh,  Sylhet, Chattogram, Barisal, Khulna, Jessore, Kustia,  2019 | 57 | 0 | 18 (Cosmopolitan) | 39 | 0 | N/A | DENV3-I (32), DENV3-III (2), DENV3-I,III (6) |
| Rahman et al, (2002)^30^ | Dhaka,  2000 | 30 | - | - | 8 | - | N/A | N/A |
| Siddiqua et al, (2018)^32^ | Dhaka,  2015-2017 | 45 | 21 | 23 | 0 | 0 | DENV1+DENV2 (1) | 0 |
| Pervin et al, (2003)^50^ | Dhaka, 2000 | 44 | 6 | 3 | 31 | 4 | N/A | N/A |
| Shirin et al, (2019)^52^ | Dhaka  2018 | 151, 9* | 13 | 62 | 47, 8* | 0 | DENV1+DENV3(7), DENV1+DENV2+DENV3 (2), DENV 2+DENV3 (16) | N/A |
| Rahim et al, (2021)^53^ | Dhaka  2017 | 161 | 7 | 147 | 7 | 0 | N/A | N/A |
|  | 2018 | 127 | 33 | 52 | 42 | 0 |  |  |
|  | 2019 | 86 | 7 | 0 | 79 | 0 |  |  |
|  | 2020 | 1 | 0 | 0 | 1 | 0 |  |  |
|  | 2021 | 178 | 0 | 0 | 178 | 0 |  |  |
| Ahmad et al, (2020)^54^ | Dhaka (2018) | 24 | 0 | 13 | 11 | 0 | N/A | Cosmopolitan, DENV3-I |

- Indicated dead participants
