# Supplementary figures and images for: Evolving epidemiology, clinical features, and genotyping of dengue outbreaks in Bangladesh, 2000–2024: a systematic review
Source: Front Microbiol. 2024 Oct 30;15:1481418. doi: 10.3389/fmicb.2024.1481418 (PMC11557403; doi:10.3389/fmicb.2024.1481418)

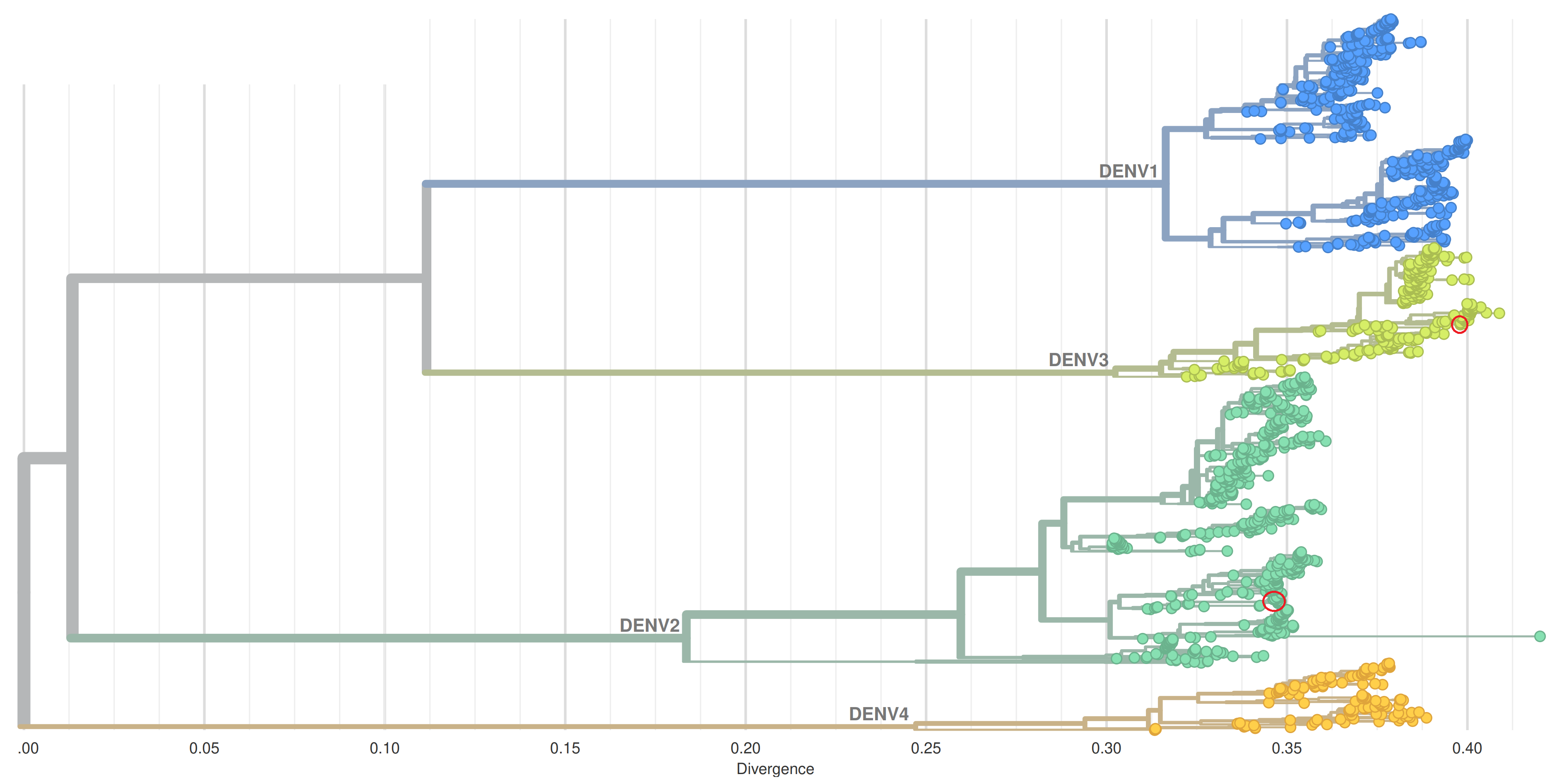

Supplement: Supplementary file 2 [file Image_1.TIF]
